# Analytical challenges in estimating the effect of exposures that are bounded by follow-up time: experiences from the Blood Stream Infection—Focus on Outcomes study

**DOI:** 10.1186/s12874-021-01393-9

**Published:** 2021-09-30

**Authors:** Rebecca Evans, Katie Pike, Alasdair MacGowan, Chris A. Rogers

**Affiliations:** 1 Bristol Trials Centre (CTEU), Bristol Medical School, University of Bristol, Level 7, Bristol Royal Infirmary, Queen’s Building, Bristol, BS2 8HW UK; 2 Infection Sciences, Pathology, North Bristol NHS Trust, Bristol, UK

**Keywords:** Bloodstream infection, Mortality, Modifiable exposure, Time-varying covariates, Immortal time bias

## Abstract

**Objective:**

To illustrate the challenges of estimating the effect of an exposure that is bounded by duration of follow-up on all-cause 28-day mortality, whilst simultaneously addressing missing data and time-varying covariates.

**Study design and methods:**

BSI-FOO is a multicentre cohort study with the primary aim of quantifying the effect of modifiable risk factors, including time to initiation of therapy, on all-cause 28-day mortality in patients with bloodstream infection. The primary analysis involved two Cox proportional hazard models, first one for non-modifiable risk factors and second one for modifiable risk factors, with a risk score calculated from the first model included as a covariate in the second model. Modifiable risk factors considered in this study were recorded daily for a maximum of 28 days after infection. Follow-up was split at daily intervals from day 0 to 28 with values of daily collected data updated at each interval (i.e., one row per patient per day).

**Analytical challenges:**

Estimating the effect of time to initiation of treatment on survival is analytically challenging since only those who survive to time t can wait until time t to start treatment, introducing immortal time bias. Time-varying covariates representing cumulative counts were used for variables bounded by survival time e.g. the cumulative count of days before first receipt of treatment. Multiple imputation using chained equations was used to impute missing data, using conditional imputation to avoid imputing non-applicable data e.g. ward data after discharge.

**Conclusion:**

Using time-varying covariates represented by cumulative counts within a one row per day per patient framework can reduce the risk of bias in effect estimates. The approach followed uses established methodology and is easily implemented in standard statistical packages.

**Supplementary Information:**

The online version contains supplementary material available at 10.1186/s12874-021-01393-9.

## Key points

- Estimating the effect of an exposure that is bounded by survival time on mortality can produce biased estimates.
- Using time-varying covariates to represent cumulative counts in a survival analysis can reduce the risk of bias in effect estimates.
- The approach described uses established methodology, so is easily implemented in standard statistical packages

## Background

Estimating the effect of time to initiation of treatment on survival is analytically challenging since it requires the person to survive until the date they receive treatment. This means that only those who survive a long time can wait a long time to start treatment and those that die shortly after start of follow-up have not had the opportunity to be exposed to a long time to initiation. This introduces a form of time-dependent bias known as immortal time bias, a bias that arises when there is a period of follow-up in which the outcome e.g. death cannot occur. In the example of time to initiation of treatment, a person who starts treatment on day 7 is considered “immortal” for the first 7 days.

Different approaches to control for the bias that arises when estimating the effect of treatment initiation on outcome have been studied in the statistical literature by several authors. Zheng Zou et al.compared methods to control for survival bias associated with treatment initiation [1]. One method classified patients into users (those who started treatment) and non-users (those who did not start treatment) at the end of follow-up. However this resulted in an overestimate of the treatment effect as patients’ future exposure was used to define the groups and therefore the event-free time in the user group was inflated. Another approach proposed was to start follow-up after a given exposure time (e.g., 90 days) allowing all patients 90 days exposure to start treatment. Patients who experience the event within the 90 days exposure are excluded, and those who do not experience the event within the 90 days are classified into users and non-users at this time and followed up from the end of this exposure time. However, this method loses a lot of study information (the first 90 days follow-up is excluded). The final approach described used a time-dependent variable for treatment which assigned the value of the treatment variable as 0 before the time of first treatment and changes to 1 when the treatment starts. For the non-user, the value remains as 0 throughout the whole follow-up. This method accurately represented the exposure status without the need to exclude participants and has shown to reduce bias in other studies [2–5]. For example, estimates from a time-dependent model provided estimates closest to the true treatment effect in a study to assess the effectiveness of postmastectomy radiation therapy in patients with cancer whilst controlling for variations in the timing of initiation of radiation therapy [6].

The aim of this paper is to illustrate the challenges of analysing an exposure that is bounded by survival time, simultaneously to other analytical challenges such as missing data and time-varying covariates through the use of a case study of an observational study investigating modifiable risk factors for mortality in bloodstream infection (BSI).

Bloodstream infection (BSI) is common in the UK and at least 100,000 patients have an episode of BSI in England, Wales and Northern Ireland each year [7]. The death rate from these infections can reach 15–25% at 30 days post-infection and 50% at 3 years depending on the pathogen involved, site of infection and other patient factors [8–10]. A number of non-modifiable patient factors (e.g. comorbidities and infection severity) are known to impact adversely on outcome [8, 11, 12]. However, to date there are no specific NHS studies exploring the impact of modifiable risk factors such as ward staffing levels, movements between wards and workload on outcomes in patients with a BSI.

Bloodstream Infections – Focus on Outcomes (BSI-FOO) was a multicentre, prospective cohort study with the primary objective of identifying modifiable risk factors associated with all-cause mortality in patients with a BSI. The study has been described in detail previously [13].

The aim of the current article is to illustrate the statistical challenges of analysing an exposure that is bounded by survival time whilst simultaneously addressing other analytical challenges such as missing data and time-varying covariates. We evaluate the use of multiple imputation, multivariable fractional polynomials and time-varying covariates; in data where having the outcome/censoring reduces the duration of exposure to the risk factors of interest.

## Methods

### Data sources

BSI-FOO was a multicentre, prospective cohort study in hospitalised adult patients with clinically significant BSI. The primary aim of the study was to quantify the effect of modifiable factors on all-cause 28-day mortality (including deaths after hospital discharge). The results of the study have been published elsewhere [13].

### Population

After excluding repeat episodes and polymicrobial infections, a total of 1,676 patients recruited from 5 centres across England and Wales were included in the study analysis. The median age was 68.5 years (interquartile range (IQR) 53.0 to 80.0) and 55% of the patients (919/1676) were male. The overall 28-day case-fatality was 20.8% (95% CI: 18.8%—22.8%).

### Follow-up

The start of follow-up began when the first positive blood sample confirming BSI was taken from the patient. This was defined as the start of the infection episode and is referred to as day 0 and time 0. Data collection ran from day 0 until day 28 or hospital discharge or death if earlier.

### Data collected on non-modifiable risk factors

Data items relating to patients’ health and care up to the start of the infection episode were considered non-modifiable. We considered all data items that were collected as part of the study which included: patient demography; date admitted to hospital; prior residence in nursing or care home; recent medical history; long-term comorbidities; measures of illness severity at or shortly before day 0; and speciality of consultant on day 0 (Table 1).

**Table 1:** Non–modifiable risk factors

Type	Factors
Organisational	Centre (5 centres) Admission from nursing- or care home Length of prior in-patient stay (days) Speciality of consultant on day 0^a^
Organism / infection	Organism identity (target organism group): 6 categories (ESBL producer, non-ESBL producing *E.coli*, MRSA, MSSA, *P. aeruginosa* and *Candida*) Source of infection (CDC criteria)
Patient measures	Age Gender Height (cm) Weight (kg)
Patient medical history (up to date 0)	Leukaemia within 5 years before date 0 Lymphoma within 5 years before date 0 Solid tumour within 5 years before date 0 Any other (second) tumour within 5 years before date 0 Chemotherapy in month before date 0 Surgery requiring overnight stay within 7 days before date 0 Burn requiring hospital admission within 7 days before date 0 Cardiac arrest within 7 days before date 0 Myocardial infarction, symptomatic within 7 days before date 0 Renal support within 7 days before date 0
Patient comorbidities ongoing at date 0	Disease markers Ascites Diabetes without organ damage Diabetes with organ damage Chronic obstructive pulmonary disease Congestive heart failure Connective tissue disease Cerebrovascular disease Dementia Hemiplegia Peptic ulcer disease Peripheral vascular disease Potentially removable sources of infection Abscess at time 0 Infected foreign body (non-surgical) at time 0 Infected prosthesis or similar surgical item at time 0
Infection severity measures at or nearest before time 0	Signs Mental Disorientation (scale 0–4) at time 0 Temperature (°C) at time 0 Systolic blood pressure (mmHg) at time 0 Early warning score at time 0 Blood tests INR^b^ at day0, or nearest within 7 days before eGFR^c^ (mL/min/1.73 m^2^) at day 0, or nearest within 7 days before Serum albumin (g/L) at day0, or nearest within 7 days before Bilirubin (total, micromol/L) at day0, or nearest within 7 days before Neutrophil count (× 10^9^/L) at day 0, or nearest within 7 days before Interventions Receiving intravenous fluids on day 0, at or before time 0 Receiving artificial ventilation on day 0, at or before time 0 Receiving vasopressor drugs on day 0, at or before time 0 Received systemic corticosteroids in 24 h before time 0

^a^ Speciality of consultant on day 0 was not included as a potential covariate in modelling as it was correlated with day 0 ward speciality, which was of more interest. ^b^*INR* international normalised ratio ^c^*eGFR* estimated glomerular filtration rate

### Data collected on modifiable risk factors

The modifiable risk factors considered were aspects of hospital care received during the follow-up and included: i) ward staffing levels, ward activity (i.e. number of admissions/discharges) and patient movements between wards; (ii) antimicrobial use (e.g. timing of start of appropriate therapy); (iii) use of intravenous lines and catheters. Ward level variables i.e. staffing levels, ward activity and movements between wards were recorded daily for each patient up to day 7 and antimicrobial use and use of intravenous lines and catheters were assessed daily up to day 28 (Table 2).

**Table 2:** Modifiable risk factors

**Risk factor**	**Definition**	**Detail**
Ward speciality^a^	Medicine, Major surgery, Minor surgery, Critical care or Other	Observed each day, days 0–7
Staffing per 10 beds (nursing and care staff)	Average number of staff (NHS-employed nurses + agency nurses + healthcare assistants) over the 3 shifts, per 10 beds	Observed each day, days 0–7
Ward activity per 10 beds	Number of patients admitted to ward + number of patients discharged from ward, per 10 beds	Observed each day, days 0–7
Central venous line	Central line present, yes or no	Determined^b^ each day, days 0–28
Peripheral vascular line	Peripheral line present, yes or no	Determined^b^ each day, days 0–28
Urinary catheter	Urinary catheter present, yes or no	Determined^b^ each day, days 0–28
Ward movement: to critical care	Cumulative count of moves from a critical care ward to a medical or surgical ward	Total number of relevant ward moves up until that day, for days 0–7
Ward movement: from critical care	Cumulative count of moves from a critical care ward to a medical or surgical ward	Total number of relevant ward moves up until that day, for days 0–7
Ward movement: within speciality	Cumulative count of ward moves within the same speciality (surgery, medicine or critical care)	Total number of relevant ward movements up until that day, for days 0–7
Ward movement: from medicine to surgery	Cumulative count of moves from a medical to a surgical ward	Total number of relevant ward movements up until that day, for days 0–7
Ward movement: from surgery to medicine	Cumulative count of moves from a surgical to a medical ward	Total number of relevant ward movements up until that day, for days 0–7
Time to initiation of appropriate antimicrobial therapy	Cumulative count of days before first receipt of appropriate antimicrobial therapy	Total number of days before first appropriate therapy up until that day, for days 0–28

^a^ For statistical modelling purposes, ward specialities were grouped as medicine, surgery (minor surgery + major surgery) and critical care; ward specialities in the “Other” category were included in either surgery (obstetrics & gynaecology) or medicine (A&E, emergency assessment, fracture clinics and related units, imaging, diagnostics and telemetry, and other services not already classified as medical, surgical or HDU/ITU). ^b^ Determined from presence/absence of line/catheter on day 0 and date of removal

Antimicrobial therapy was defined as ‘appropriate’ if the organism was susceptible to the antimicrobial prescribed and the therapy was continued for at least 36 hours [13, 14]. Consecutive appropriate antimicrobials were treated as a single period of appropriate therapy, provided that the subsequent therapy began within 24 h of the last dose of the previous therapy [13].

### Analysis approach

The primary analysis involved building two Cox proportional hazards models, one for non-modifiable risk factors and one for the modifiable risk factors.

Firstly, a Cox proportional hazards model was fitted with an outcome of death within 28 days of BSI and the non-modifiable risk factors as explanatory variables. The risk factors considered for inclusion in the model were all time-invariant (i.e. measured at one point in time) and are given in Table 1, with factors included in the final multivariable model identified using backwards selection with a 20% significance level to ensure the risk score encompassed the most important non-modifiable risk factors for mortality. This model was used to derive a risk score for each patient. Then, the second Cox proportional hazards model was fitted with an outcome of death within 28 days of BSI and the modifiable risk factors (Table 2) and risk score derived from the first model included as covariates. In this second model all modifiable risk factors were included regardless of statistical significance as they were all of clinical interest. In addition, the following pre-specified interaction terms were considered for potential inclusion in the model—organism by: risk score, presence of central line, presence of peripheral line, presence of urinary catheter, time to appropriate antimicrobial therapy; and ward specialty by: ward activity, ward staffing levels, within-ward speciality movements. A forward stepwise approach was taken to select which interactions were to be included in the final model, using likelihood ratio tests and 10% significance levels to compare nested models. The model selection process was performed on a single randomly selected imputed dataset so that log-likelihood statistics could be calculated and compared. Interaction terms between ward speciality and: a) ward activity and b) staffing levels, were included in the model regardless of statistical significance to allow estimation of effects within each ward specialty.

Schoenfeld residuals and log–log plots of survival were used to assess the proportional hazards assumption [15]. If the assumption was not met, time was categorised into periods where proportional hazards appeared valid, and the effect of the variable causing non-proportional hazards estimated separately for each of the categorised time periods. Collinearity was examined using the variance inflation factor with values < 5 considered acceptable [16].

## Analysis challenges

The analysis presented several challenges, with the added complexity that they needed to be addressed simultaneously. Analysis challenges and how they were addressed are discussed below:

### Missing data (multiple imputation)

Individual data items were missing for between 10 and 45% of patients. Fully conditional specification (FCS) multiple imputation was used to impute missing data, under the assumption that data was missing at random. Imputation by chained equations, an iterative procedure to generate imputed values, was used to generate multiple complete data sets (using Stata’s -ice- command). All variables that were in the primary analysis models (modifiable and non-modifiable), variables predictive of missingness or the value of variables with missing data (Table 1), indicator for death and the log of survival time were included in the imputation procedure [17]. Some of the daily collected variables were not applicable after discharge/death e.g. ward variables. This was incorporated into the imputation model by transposing the data into wide format (“one row per patient”) and using conditional imputation. Ward variables for each day were imputed conditional on patients being alive and in hospital on that day, using data from the other ward variables on that day only, and using data from the variable itself on all other days. For example, ward activity on day 3 was imputed (conditional on the patient being alive and in hospital on day 3) using ward activity on all other days, staffing on day 3, ward speciality on day 3, and ward movements on day 3.

Non-normally distributed variables were transformed prior to imputation, with the most suitable transformation being selected using Stata’s -gladder- command. If a suitable transformation could not be found or the imputation procedure imputed values outside valid ranges, then predictive mean matching was used for the imputation of that variable. The number of imputations, m, was set to be equal to the value of the percentage of missing data for the variable with the highest proportion of missing data (m = 45) [18].

Within all analysis models, Rubin’s rule was used to summarise data across the imputed datasets [19].

### Deriving a risk score for non-modifiable risk factors

In deriving the risk score representing the non-modifiable risk factors, it was important to ensure the most suitable transformation of each continuous covariate was used in order to provide the best summary of the relationship between the non-modifiable factors and mortality. Also, interpretation of the estimates for each factor was not required. Therefore, multivariable fractional polynomial models were fitted on the imputed dataset, within a Cox proportional hazards model (using -mfpmi- Stata command) [20, 21]. This iterative procedure includes backwards selection steps to select variables that are predictive of mortality (threshold for variable selection was set as alpha = 0.2), whilst finding the most suitable functional form of such covariates (maximal polynomial degree 2 i.e. FP2 models), within a time-to-event framework e.g. fitting the non-modifiable risk factor model using fractional polynomials resulted in systolic blood pressure (SBP) being transformed using the equation ((SBP/100)^-2)-0.68.

The model was fitted for the cohort as a whole and the model estimates were used to derive a risk score for each patient.

### Accounting for daily variation in ward variables

**Fig. 1:**
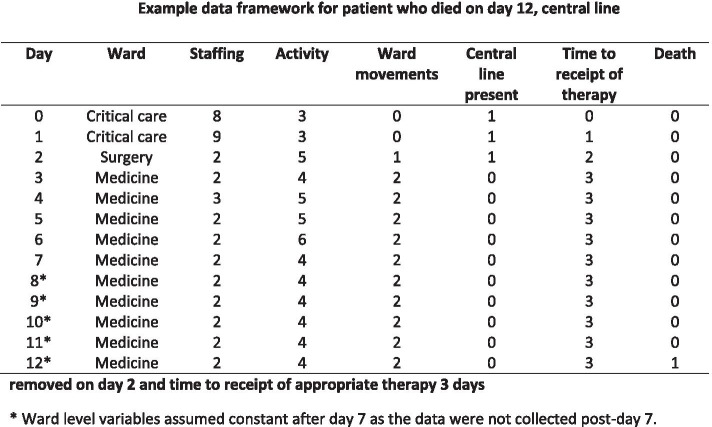
Example data framework for patient who died on day 12, central line removed on day 2 and time to receipt of appropriate therapy 3 days. * Ward level variables assumed constant after day 7 as the data were not collected post-day 7

To account for the daily variation in ward speciality, ward staffing and ward activity duration of follow-up was split (using -stsplit- Stata command) at daily intervals from day 0 to 28 and variables were represented by time-varying covariates. The “stsplit” command within Stata splits each observation into multiple records on the basis of analysis time e.g. one record per day. Ward speciality, presence of central line, presence of peripheral line, presence of urinary catheter, ward movements, ward staffing levels, ward activity, and antimicrobial therapy variable values were updated at each interval. For patients who survived and were not discharged prior to day 7, ward variables and ward movements for the remaining days (day 7 up to day 28 or death/discharge) were assumed to be constant for this period as the data were not collected post-day 7 and it was not anticipated that many patients would move wards after day 7. See Fig. 1 for example data framework for patient who died on day 12, central line removed on day 2 and time to receipt of appropriate therapy 3 days.

### Dealing with variables that are bounded by survival time

Estimating the effect of time to initiation of treatment on survival is analytically challenging since it requires the person to survive until the day they receive treatment. This means that only those who survive to time t can wait until time t to start treatment and those that die shortly after start of follow-up have not had the opportunity to be exposed to a long time to initiation. This introduces immortal time bias, a type of time-dependent bias that arises when there is a period of follow-up in which the outcome (e.g. death) cannot occur [1, 22]. In the example of time to initiation of treatment, a person who starts treatment on day 7 is considered “immortal” for the first 7 days.

Only mortality data was collected post-discharge, so patients who were discharged/died during the 28-day follow-up had less data available than those in hospital for a longer duration. Ward specialty was determined at a single time point each day, so the maximum number of ward movements recorded was one per day in hospital. This meant that the total number of movements between wards was bounded by the number of days a patient remained alive and in hospital. Therefore, using total number of ward movements as a covariate in the model could lead to misleading or biased results as patients who died earlier were at “risk” of moving wards for a shorter period of time compared to patients who survived for a longer period. For example, if a patient died on day 3, the maximum number of ward movements they could have experienced was 3, compared to 7 movements for a patient who died on day 7. To account for this in the model, we planned to use time-varying covariates to represent a cumulative count of moves within the “stsplit” framework. For each day at risk, the ward movement count was increased by one if the patient moved wards or remained the same if the patient did not move wards. However, due to small number of patients with more than one ward movement, ward movement was included as a binary variable (one or more vs none) in the model, treated as a time-varying covariate (0 until the patient moves wards, then updated to 1 on day of their first ward movement). Additionally, some ward movements could have been related to improving patient condition, and some could have related to deteriorating condition. To account for this, we investigated the cumulative count of ward movements split by type of ward movement. Types of ward movements considered were: movement to critical care, movement from critical care, movement within ward specialty, movement from medicine to surgery, and movement from surgery to medicine.

Similarly, time to appropriate therapy was also bounded by survival time. We therefore used time-varying covariates to represent cumulative count of days before first receipt of appropriate antimicrobial therapy, instead of total number of days until receipt of therapy. That is, on day 0 time to receipt of appropriate therapy was 0 for all patients, remaining at 0 each day for patients who received appropriate therapy on day 0 otherwise increasing by one for each additional day until receipt of first appropriate therapy. This ensured that for each day, the maximum number of days until initiation of therapy did not exceed the number of days under analysis for both those who died and those who survived.

### Proportional hazards assumption

Residual checks suggested that inclusion of the time to receipt of appropriate antimicrobial therapy violated the proportional hazards assumption. Time to receipt of appropriate antimicrobial therapy appeared to have a greater impact on mortality during the first 7 days and the proportional hazard assumption appeared valid (upon examination of the plots of cumulative incidence) separately within each of the intervals between day 0 and 7, day 7 and 14 and day 14 and 28. Therefore time was categorised into these intervals and the model fitted with an interaction between these time intervals and time to appropriate antimicrobial therapy. After fitting the model with this interaction, the proportional hazards assumption was met.

### Impact of the 36-h rule in the definition of appropriate therapy

By definition, an antimicrobial treatment would not be considered ‘appropriate’ if the patient died within 36 h of starting it, since they need to have received the therapy for at least 36 h for it to be defined as appropriate. This may lead to deaths within 36 h being viewed as a consequence of not receiving the therapy, when in fact the patient was in receipt of therapy but (due to death) the therapy was not administered for the 36 h required to be defined as appropriate. We performed a sensitivity analysis with the “36-h rule” removed to assess this possibility.

## Study results

A summary of missing data is given in Supplementary Table 1, Additional file 1 and the model used to derive the risk score is shown in Supplementary Table 2, Additional file 1. After adjustment for organism and the risk score representing the non-modifiable risk factors, modifiable risk factors that were associated with mortality within 28 days were ward speciality, ward activity, cumulative count of ward movements within speciality, cumulative count of movements from critical care, and time to receipt of appropriate therapy [13]. Inclusion of time to receipt of appropriate antimicrobial therapy violated the proportional hazards assumption, therefore the effect of time to receipt of appropriate antimicrobial therapy was estimated separately within each of three intervals: days 0–6, days 7–13, and day14 onwards. During the first week (days 0–6 inclusive), there was a highly significant effect for all organisms. After the first week, for patients who survived to day 7, the effect of time to receipt of first appropriate therapy on 28-day mortality was not statistically significant [13]. The final model can be seen in Supplementary Table 3 and Supplementary Figure 1, Additional file 1 and further details published in the results manuscript [13]. The complete case analysis gave similar results regarding the interest parameters and is shown in Supplementary Figure 2, Additional file 1. In the sensitivity analysis with the “36-h rule” removed, the effect of organism was more strongly associated with mortality, and the effect of time to appropriate antimicrobial therapy within the first week (days 0–6) was less strongly associated with mortality compared to the primary analysis (Supplementary Figure 3, Additional file 1). This sensitivity analysis was also performed with a “12-h rule” and “24-h rule” which showed similar effects to when using the 36-h rule (Supplementary Figures 4 and 5, Additional file 1).

## Discussion

This paper highlights the complexity of addressing several analytical issues simultaneously; multiple imputation; multivariable fractional polynomials; time-varying covariates; and immortal time bias. In particular, it presents a method which is easily implemented within a Cox proportional hazard model to deal with data where the exposure is bounded by survival time.

Multiple imputation using chained equations was used to impute missing data. A large number of data items were collected in the study which enabled us to include variables that were predictive of missing data in a covariate of interest in the multiple imputation procedure. We therefore deem the missing at random assumption plausible. Conditional imputation can be used to impute longitudinal data, e.g. ward variables for each day were imputed conditional on patients being alive and in hospital on that day, using data from other ward variables on the other days. An alternative approach to imputing longitudinal data is two-fold imputation [23]. However, this requires numerous iterations in each time period which makes it computationally intensive. It has also been shown that the two-fold method produces slightly more biased and less precise estimates than the standard approach [24, 25].

Within the imputation procedure, we had planned to allow for any interaction terms that were included the main analysis model by running the imputation procedure separately for each organism or ward specialty (depending on the variable(s) was involved in the interaction). Unfortunately, computational problems prevented this. Models including the six categories of organism type did not converge, while the time-varying nature of ward speciality made it difficult to choose a time point at which to split the dataset in order to perform the imputation on a “one row per patient” model; data was also missing in ward speciality itself.

To account for risk factors which can vary across the study period, follow-up can be split at intervals with risk factors being updated at each interval and included in the model as time-varying covariates. Where risk factors are bounded by the outcome, a cumulative count can be used within the “stsplit” framework to help overcome this. This ensures that, for each day of risk, the maximum number of days exposed/unexposed does not exceed the time at risk. This enabled us to reduce the risk of immortal time bias, and can also be used when estimating the effect of duration of treatment on survival. A common approach to estimate the effect of a variable with non-proportional hazards is to allow time-varying effects by including an interaction between the variable of interest and some function of time. In this study time to appropriate therapy violated the proportional hazards assumption, however the proportional hazards assumption was met for short time periods and the time-varying effect could be adequately captured with time split into the 3 categories (0–6 days, 7–14 days and > 14 days). However, caution needs to be taken when interpreting time varying period specific hazard ratios due the potential ‘built in’ selection bias [26] i.e. the calculation of the hazard ratio for period t1 to t2 is restricted to people who survive to time t1 and they may be a select cohort of the population at time 0. For example, in a randomised controlled trial, a subset of the population may have an unmeasured confounder, X, which at baseline should be balanced by the treatment allocation. However, when calculating time specific hazard ratios e.g. for the time period t2-t3, the analysis is restricted to patients who survive beyond time t2. This confounder (X) may be unbalanced at that time with respect to the treatment allocation if survival is affected by both the unmeasured confounder (X) and treatment allocation [27, 28]. This could bias the results. Effect estimates should therefore be interpreted as associations and not causal effects.

There was concern about the possibility of reverse causation in the conclusions of appropriate therapy, as it was predefined as treatment for at least 36 h with an antimicrobial to which the organism was susceptible. This meant that deaths within 36 h of a first dose of suitable antimicrobial could be associated with a lack of appropriate therapy and therefore strengthen the apparent effect of receiving appropriate therapy on survival. We therefore repeated the analysis of 28-day mortality with 24-h, 12-h and 0-h rules in place of the 36-h rule. Compared to the 36-h rule, the apparent impact of time to appropriate therapy was reduced slightly with the 24-h rule and slightly more so with the 12-h rule. These results are consistent with reverse causation inflating the estimated effect, and shorter defined minimum periods reduced the extent of this. The 0-h rule, however, gave quite different estimates with the estimated impact of time to appropriate therapy being greatly reduced, though we deemed this not to be a true reflection of appropriate therapy. Patients who die very soon after starting therapy are likely to have not received the treatment long enough for it to take effect and therefore it is unsurprising that the effect of time to receipt of truly appropriate therapy is highly diluted.

Applying these methods enabled us to determine that ward speciality, ward activity, ward movement within speciality, movements from critical care, and time to receipt of appropriate antimicrobial were all risk factors associated with mortality within 28 days. Using cumulative counts within a one row per day framework in a survival analysis can reduce the risk of bias. The approach that we followed uses already established methodology, so it is easily implemented in standard statistical packages, including Stata.

## Supplementary Information

**Additional file 1.**

## Data Availability

The datasets used and/or analysed during the current study are available from the corresponding author on reasonable request.

## Abbreviations

BSI: Bloodstream infection
BSI-FOO: Bloodstream Infections – Focus on Outcomes
CI: Confidence interval
IQR: Interquartile range
NHS: National Health Service
SBP: Systolic blood pressure
UK: United Kingdom
