# Supplementary material for: Analytical challenges in estimating the effect of exposures that are bounded by follow-up time: experiences from the Blood Stream Infection—Focus on Outcomes study
Source: BMC Med Res Methodol. 2021 Sep 30;21:197. doi: 10.1186/s12874-021-01393-9 (PMC8482664; doi:10.1186/s12874-021-01393-9)
Supplement: Supplementary file 1 — Additional file 1. [file 12874_2021_1393_MOESM1_ESM.pdf]

Analytical challenges in estimating the effect of exposures that are bounded by follow-up time: Experiences from the Blood Stream Infection - Focus on Outcomes study

Additional file 1

Supplemental table 1 Summary of missing data, by 28-day survival status....................................... 2
Supplemental table 2 Multivariable Cox model of non-modifiable risk factors on 28-day mortality 4
Supplemental table 3 Primary outcome - modifiable risk factors on 28 day mortality..................... 6

Supplemental figure 1 Modifiable risk factors on 28 day mortality 8
Supplemental figure 2 Sensitivity analysis of primary outcome: Complete case analysis 9
Supplemental figure 3 Sensitivity analysis of primary outcome: removal of "36 hour rule" in definition of time to appropriate therapy 10
Supplemental figure 4 Sensitivity analysis of primary outcome: 12-hour (in place of 36-hour) rule in definition of time to appropriate therapy 11
Supplemental figure 5 Sensitivity analysis of primary outcome: 24-hour (in place of 36-hour) rule in definition of time to appropriate therapy 12

Supplemental table 1 Summary of missing data, by 28-day survival status

Risk factor	Survived (n=1,328)		Died (n=348)		Overall (n=1,676)	
	n missing	% missing	n missing	% missing	n missing	% missing
**Patient measures**						
Age	0	0.0%	0	0.0%	0	0.0%
Male	0	0.0%	0	0.0%	0	0.0%
Body mass index	604	45.5%	195	56.0%	799	47.7%
**Patient medical history**						
Chemotherapy in month before date 0	0	0.0%	0	0.0%	0	0.0%
Any tumour within last 5 years	0	0.0%	0	0.0%	0	0.0%
Surgery (overnight stay) ≤7 days before date 0	1	0.1%	0	0.0%	1	0.1%
Burn requiring admission ≤7 days before date 0	2	0.2%	1	0.3%	3	0.2%
Cardiac arrest ≤7 days before date 0	0	0.0%	0	0.0%	0	0.0%
Renal support ≤7 days before date 0	0	0.0%	0	0.0%	0	0.0%
Myocardial infarction ≤7 days before date 0	0	0.0%	0	0.0%	0	0.0%
**Infection severity measures**						
Mental Disorientation	1	0.1%	0	0.0%	1	0.1%
Temperature (°C) at time 0	20	1.5%	10	2.9%	30	1.8%
eGFR (mL/min/1.73m^2)	98	7.4%	20	5.7%	118	7.0%
Serum albumin (g/L)	161	12.1%	39	11.2%	200	11.9%
Bilirubin total (micromol /L)	216	16.3%	51	14.7%	267	15.9%
Neutrophil count at day 0 or closest (x10^9/L)	110	8.3%	29	8.3%	139	8.3%
Systolic BP at day 0 or closest (mmHg)	196	14.8%	50	14.4%	246	14.7%
On intravenous fluids at day 0	4	0.3%	0	0.0%	4	0.2%
On ventilation at day 0	5	0.4%	0	0.0%	5	0.3%
On vasopressor drugs at day 0	1	0.1%	0	0.0%	1	0.1%
Systemic corticosteroids in last 24 hours	4	0.3%	1	0.3%	5	0.3%
**Patient comorbidities at date 0**						
Congestive heart failure	0	0.0%	0	0.0%	0	0.0%

**Risk factor**	**Survived (n=1,328)**		**Died (n=348)**		**Overall (n=1,676)**	
	**n missing**	**% missing**	**n missing**	**% missing**	**n missing**	**% missing**
Peripheral vascular disease	0	0.0%	0	0.0%	0	0.0%
Cerebrovascular disease	0	0.0%	0	0.0%	0	0.0%
Hemiplegia	0	0.0%	0	0.0%	0	0.0%
Dementia	1	0.1%	0	0.0%	1	0.1%
Chronic obstructive pulmonary disease	1	0.1%	0	0.0%	1	0.1%
Connective tissue disease	0	0.0%	0	0.0%	0	0.0%
Peptic ulcer disease	0	0.0%	0	0.0%	0	0.0%
Ascites	0	0.0%	0	0.0%	0	0.0%
Diabetes	0	0.0%	0	0.0%	0	0.0%
Infected foreign body at time 0	1	0.1%	1	0.3%	2	0.1%
Source of infection (CDC criteria)	1	0.1%	0	0.0%	1	0.1%
**Organisational factors**						
Admission from nursing home	1	0.1%	0	0.0%	1	0.1%
Length of prior hospital stay (days)	0	0.0%	0	0.0%	0	0.0%
Hospital or community acquired infection	0	0.0%	0	0.0%	0	0.0%
Speciality of consultant on day 0	111	8.4%	15	4.3%	126	7.5%
Central line present at time 0	2	0.2%	1	0.3%	3	0.2%
Peripheral line present at time 0	2	0.2%	1	0.3%	3	0.2%
Urinary catheter present at time 0	2	0.2%	1	0.3%	3	0.2%

Abbreviations: eGFR=Estimated glomerular filtration rate, BP=Blood pressure, CDC= Centres for Disease Control and Prevention

Supplemental table 2 Multivariable Cox model of non-modifiable risk factors on 28-day mortality [a]

Risk factor		Hazard ratio	95% CI	P value
Age (years)		1.02	(1.01, 1.03)	<0.001
Temperature at time 0 (°C)		0.83	(0.75, 0.92)	<0.001
Weight (kg)		0.99	(0.98, 1.00)	0.136
Systolic BP at day 0 or closest (mmHg)		1.43	(1.17, 1.75)	<0.001
Admission from nursing home		1.36	(0.94, 1.98)	0.104
Serum albumin (g/L)		0.94	(0.92, 0.96)	<0.001
Bilirubin total (µmol/L)		1.00	(1.00, 1.00)	0.188
Renal support within 7 days before date 0		1.53	(1.01, 2.31)	0.044
On ventilation at day 0		1.33	(0.94, 1.90)	0.111
On intravenous fluids at day 0		1.21	(0.96, 1.53)	0.105
Systemic corticosteroids in last 24 hours		1.75	(1.35, 2.28)	<0.001
Abscess at time 0		0.63	(0.34, 1.15)	0.133
Congestive heart failure		1.35	(1.00, 1.84)	0.053
Peripheral vascular disease		1.50	(1.06, 2.12)	0.021
Cerebrovascular disease		1.32	(1.00, 1.74)	0.050
Peptic ulcer disease		1.38	(0.93, 2.05)	0.108
Ascites		2.04	(1.33 ,3.12)	0.001
Leukaemia within last 5 years		2.01	(1.36, 2.96)	<0.001
Solid tumour within last 5 years		1.38	(1.07, 1.79)	0.015
Any other tumour within last 5 years		1.54	(0.97, 2.45)	0.070
Mental disorientation:	*None*	1	-	
	Grade I	0.89	(0.35, 1.44)	
	Grade II	1.54	(1.08, 2.19)	0.136
	Grade III	1.01	(0.61, 1.67)	
	Grade IV	1.40	(0.64, 3.06)	
eGFR	*Normal/Stage 1*	1	-	0.007
	Stage 2 CKD[1]	0.94	(0.66, 1.33)	

	Stage 3 CKD	0.88	(0.61, 1.27)	
	Stage 4 CKD	1.55	(1.06, 2.26)	
	Stage 5 CKD	0.82	(0.48, 1.42)	
Source of infection	*Gastrointestinal system*	1	-	
	Line	1.62	(0.83, 3.15)	
	Lower respiratory tract	4.71	(2.58, 8.58)	
	Skin and surgical site	1.73	(0.89, 3.37)	<0.001
	Systemic & site uncertain	3.37	(1.96, 5.78)	
	Urinary tract	1.29	(0.73, 2.30)	
	Other	1.56	(0.74, 3.30)	

[a] Model adjusted for centre

Abbreviations: BP=Blood pressure, eGFR=Estimated glomerular filtration rate, CKD=Chronic kidney disease

Supplemental table 3 Primary outcome - modifiable risk factors on 28 day mortality

Variable			Hazard ratio (95% CI)	P-value
Risk score		Within medicine	2.77 (2.35 – 3.27)	<0.001
		Within critical care	1.84 (1.54 – 2.19)	<0.001
		Within surgery	2.89 (2.13 – 3.90)	<0.001
Organism [a]	*Non-ESBL E. coli*		*Reference*	0.369
	ESBL producer		1.10 (0.70 – 1.73)	
	Candida		0.57 (0.32 – 1.02)	
	MRSA		1.23 (0.74 – 2.04)	
	MSSA		1.40 (1.00 – 1.95)	
	P. aeruginosa		1.20 (0.83 – 1.74)	
Ward speciality [b]	*Medicine*		*Reference*	<0.001
	Critical care		2.11 (1.30 – 3.41)	
	Surgery		0.63 (0.39 – 1.02)	
Central line present			1.08 (0.75 – 1.56)	0.674
Peripheral line present			1.12 (0.77 – 1.63)	0.562
Urinary catheter present			1.14 (0.84 – 1.56)	0.394
Average number of staff across the 3 shifts, per 10 beds		Within medicine	1.00 (0.86 – 1.16)	0.982
		Within critical care	0.99 (0.96 – 1.02)	0.580
		Within surgery	0.95 (0.63 – 1.44)	0.810
Ward activity (number of admissions and discharges) per 10 beds		Within medicine	1.04 (1.01 – 1.06)	0.001
		Within critical care	1.12 (1.06 – 1.19)	<0.001
		Within surgery	1.03 (0.91 – 1.16)	0.643
Movement within ward speciality	One or more vs none		0.67 (0.48 – 0.93)	0.016
Movement to critical care	One or more vs none		1.32 (0.84 – 2.07)	0.228
Movement from critical care	One or more vs none		0.52 (0.24 – 1.11)	0.090
Movement from surgery to medicine	One or more vs none		1.22 (0.45 – 3.33)	0.692
Movement from medicine to surgery	One or more vs none		0.93 (0.39 – 2.20)	0.868

Time to appropriate therapy (days)	days 0–6	Within non-ESBL *E. coli*	1.45 (1.15 – 1.83)	0.001
		Within ESBL producer	1.84 (1.43 – 2.37)	<0.001
		Within *Candida*	1.62 (1.27 – 2.05)	<0.001
		Within MRSA	1.39 (1.03 – 1.88)	0.033
		Within MSSA	2.02 (1.71 – 2.38)	<0.001
		Within *P. aeruginosa*	1.69 (1.42 – 2.01)	<0.001
	days 7–13	Within non-ESBL *E. coli*	0.87 (0.68 – 1.11)	0.268
		Within ESBL producer	1.13 (0.98 – 1.31)	0.102
		Within *Candida*	0.94 (0.79 – 1.12)	0.522
		Within MRSA	1.05 (0.92 – 1.21)	0.475
		Within MSSA	0.97 (0.78 – 1.20)	0.754
		Within *P. aeruginosa*	0.74 (0.48 – 1.13)	0.166
	days 14–28	Within non-ESBL *E. coli*	0.60 (0.29 – 1.23)	0.160
		Within ESBL producer	0.93 (0.71 – 1.22)	0.587
		Within *Candida*	1.03 (0.97 – 1.11)	0.318
		Within MRSA	0.95 (0.82 – 1.11)	0.541
		Within MSSA	1.06 (0.99 – 1.14)	0.100
		Within *P. aeruginosa*	1.02 (0.95 – 1.09)	0.655

Notes:

[a] Effect of organism is given for the time period 0 to 6 days, when time to appropriate therapy is 1 day

[b] Effect of ward speciality is given for the median number of staff per 10 beds, median ward activity and median risk score

Supplemental figure 1 Modifiable risk factors on 28 day mortality

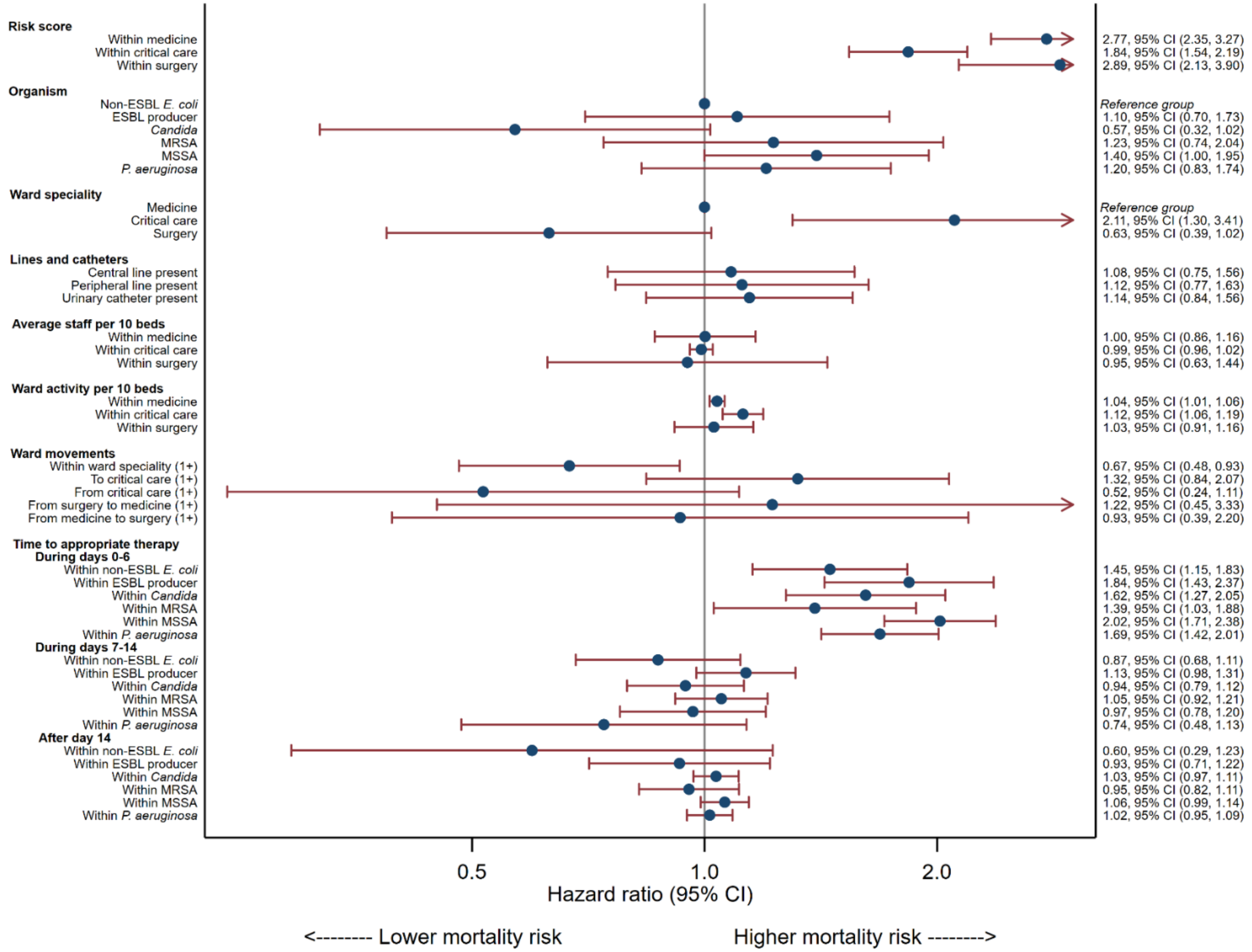

[a] Effect of organism is given for the time period 0 to 6 days, when time to appropriate therapy is 1 day

[b] Effect of ward speciality is given for the median number of staff per 10 beds, median ward activity and median risk score

Abbreviations: ESBL= Extended-spectrum beta-lactamase, MRSA- Methicillin-resistant S. aureus, MSSA= Methicillin-susceptible S. aureus, CI=Confidence interval

Supplemental figure 2 Sensitivity analysis of primary outcome: Complete case analysis

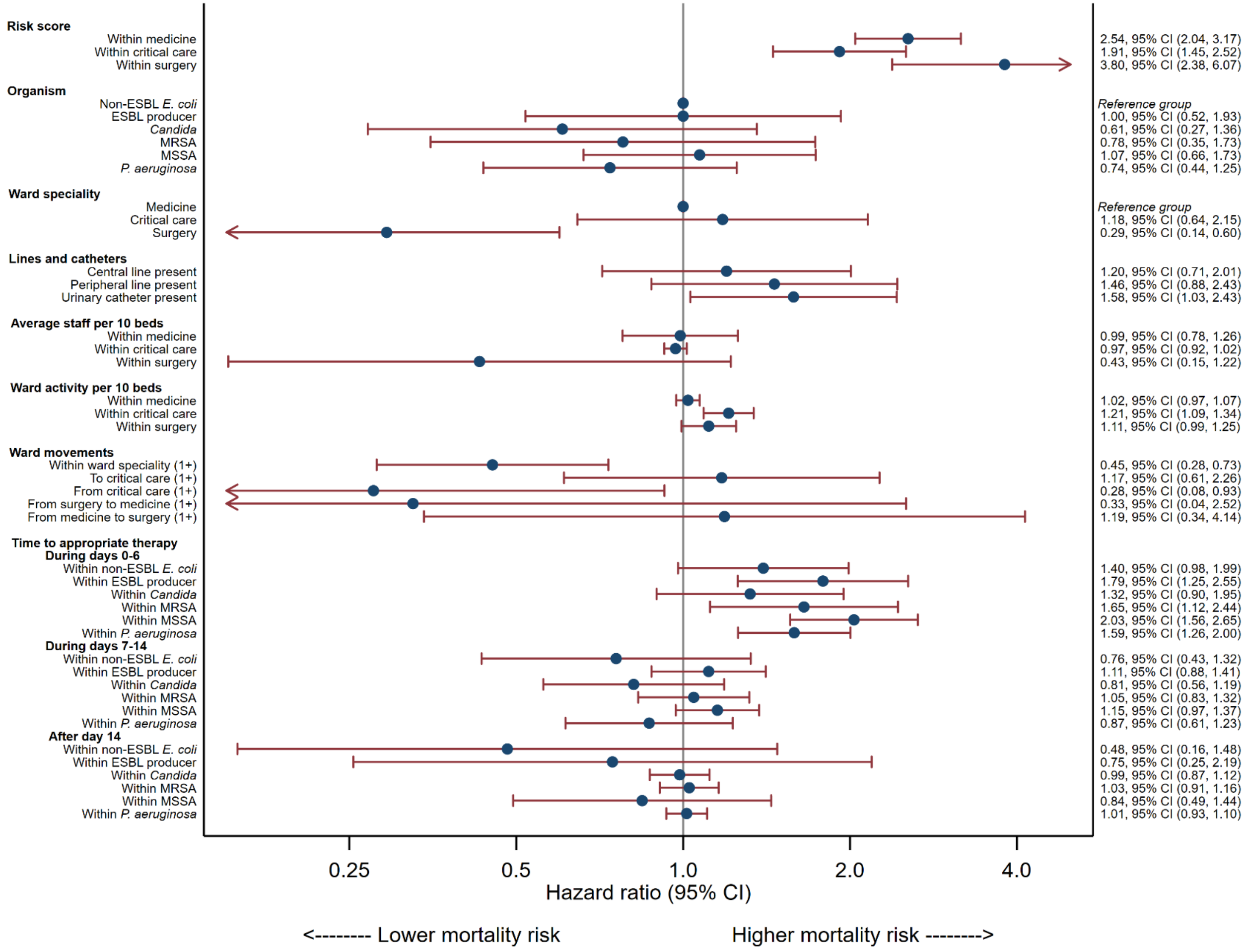

[a] Effect of organism is given for the time period 0 to 6 days and when time to appropriate therapy is 1 day

[b] Effect of ward speciality is given for the median number of staff per 10 beds, median ward activity and median risk score

Abbreviations: ESBL= Extended-spectrum beta-lactamase, MRSA- Methicillin-resistant S. aureus, MSSA= Methicillin-susceptible S. aureus, CI=Confidence interval

Supplemental figure 3 **Sensitivity analysis of primary outcome: removal of "36 hour rule" in definition of time to appropriate therapy**

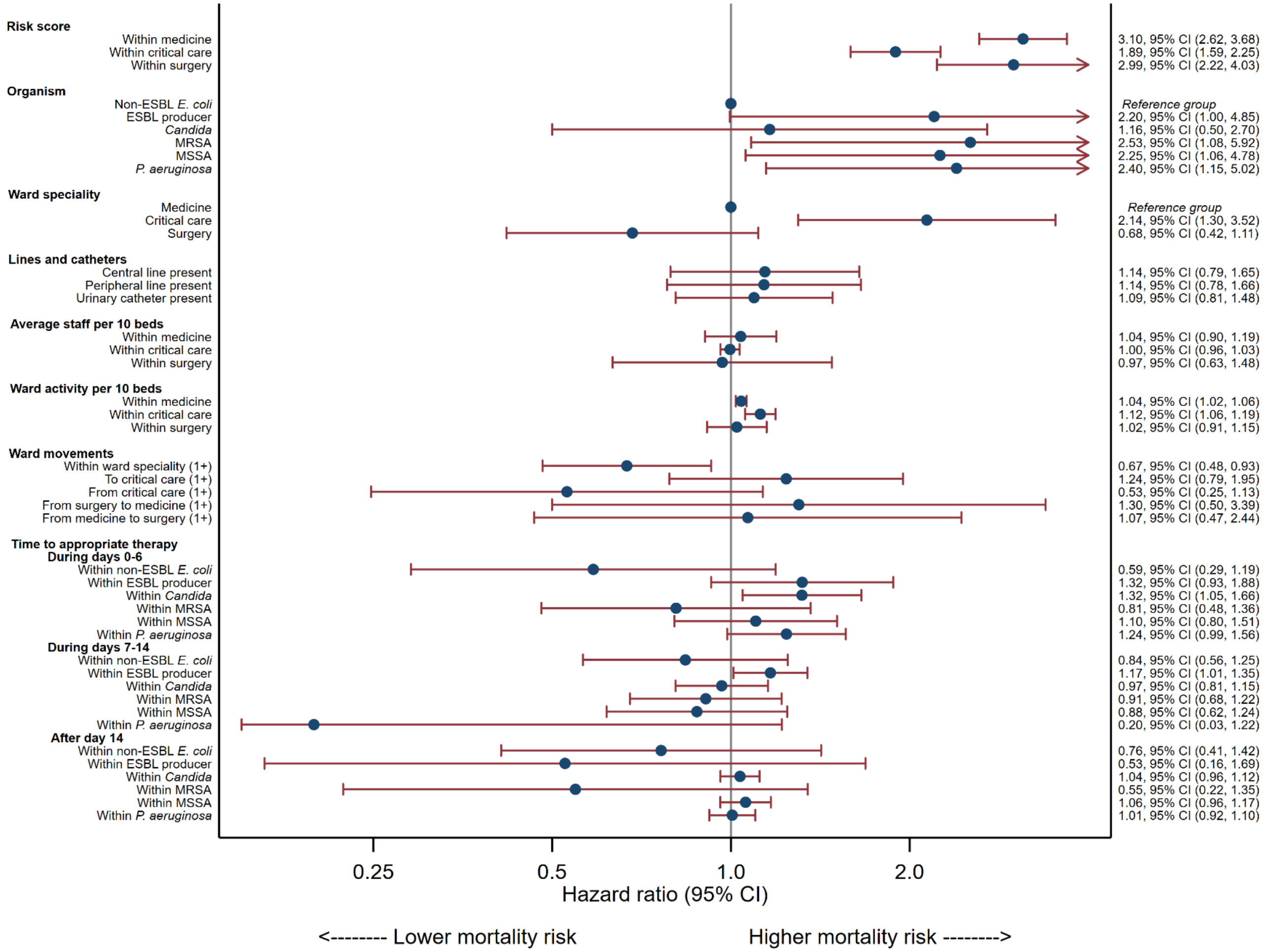

[a] Effect of organism is given for the time period 0 to 6 days and when time to appropriate therapy is 1 day

[b] Effect of ward speciality is given for the median number of staff per 10 beds, median ward activity and median risk score

Abbreviations: ESBL= Extended-spectrum beta-lactamase, MRSA- Methicillin-resistant S. aureus, MSSA= Methicillin-susceptible S. aureus, CI=Confidence interval

Supplemental figure 4 Sensitivity analysis of primary outcome: 12-hour (in place of 36-hour) rule in definition of time to appropriate therapy

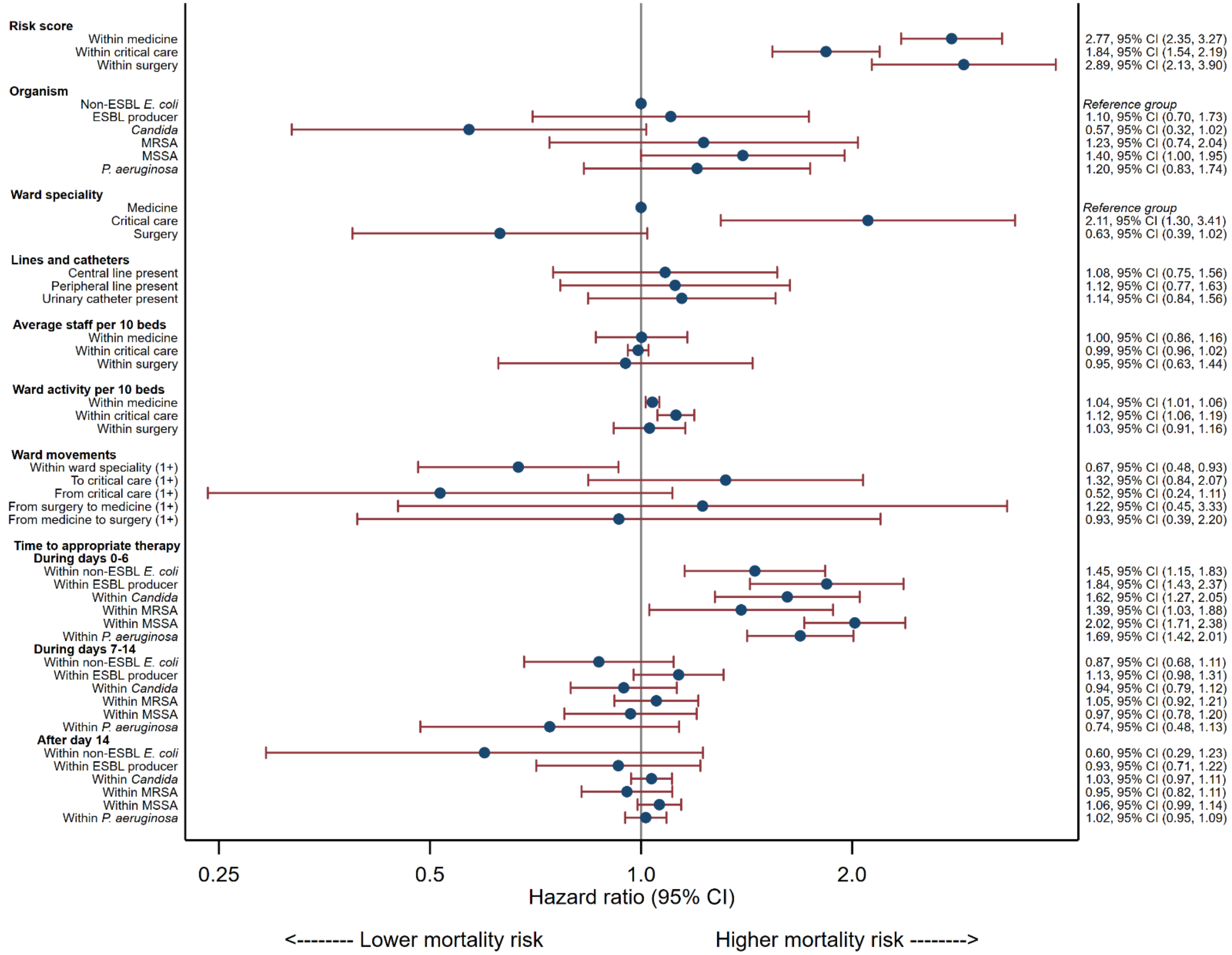

[a] Effect of organism is given for the time period 0 to 6 days and when time to appropriate therapy is 1 day

[b] Effect of ward speciality is given for the median number of staff per 10 beds, median ward activity and median risk score

Abbreviations: ESBL= Extended-spectrum beta-lactamase, MRSA- Methicillin-resistant S. aureus, *MSSA= Methicillin-susceptible S. aureus, CI=Confidence interval*

Supplemental figure 5 Sensitivity analysis of primary outcome: 24-hour (in place of 36-hour) rule in definition of time to appropriate therapy

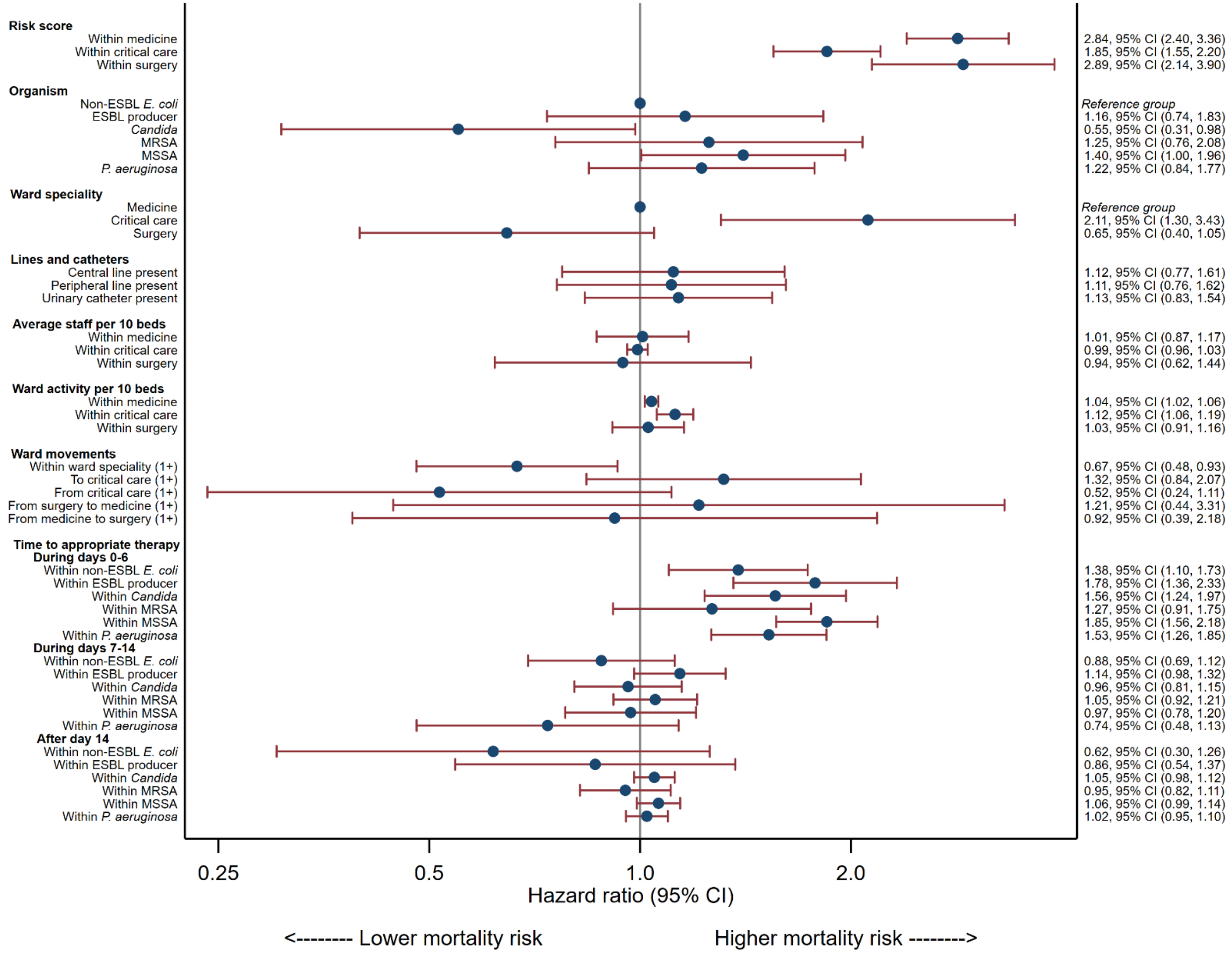

[a] Effect of organism is given for the time period 0 to 6 days and when time to appropriate therapy is 1 day

[b] Effect of ward speciality is given for the median number of staff per 10 beds, median ward activity and median risk score

Abbreviations: ESBL= Extended-spectrum beta-lactamase, MRSA- Methicillin-resistant S. aureus, MSSA= Methicillin-susceptible S. aureus, CI=Confidence interval
